# Beyond the androgen receptor: New approaches to treating metastatic prostate cancer. Report of the 2013 Prouts Neck Prostate Cancer Meeting

**DOI:** 10.1002/pros.22753

**Published:** 2013-11-19

**Authors:** Kenneth J Pienta, Guneet Walia, Jonathan W Simons, Howard R Soule

**Affiliations:** 1 Department of Urology, The James Buchanan Brady Urological InstituteBaltimore, Maryland; 2 Department of Oncology, The Johns Hopkins School of MedicineBaltimore, Maryland; 3 Department of Pharmacology and Molecular Sciences, The Johns Hopkins School of MedicineBaltimore, Maryland; 4 Prostate Cancer FoundationSanta Monica, California

**Keywords:** tumor microenvironment, metastases, diagnostics, therapeutics, treatment resistance

## Abstract

**INTRODUCTION:**

The Prouts Neck Meetings on Prostate Cancer began in 1985 through the efforts of the Organ Systems Branch of the National Cancer Institute to stimulate new research and focused around specific questions in prostate tumorigenesis and therapy.

**METHODS:**

These meetings were think tanks, composed of around 75 individuals, and divided equally between young investigators and senior investigators. Over the years, many new concepts related to prostate cancer resulted from these meetings and the prostate cancer community has sorely missed them since the last one in 2007.

**RESULTS:**

We report here the first of a new series of meetings. The 2013 meeting focused on defining how the field of treatment for metastatic prostate cancer needs to evolve to impact survival and was entitled: “Beyond AR: New Approaches to Treating Metastatic Prostate Cancer.” As castrate resistant prostate cancers escape second generation anti-androgen agents, three phenotypes/genotypes of CRPC appear to be increasing in prevalence and remain resistant to treatment: NeuroEndocrine Prostate Cancer, Persistent AR—Dependent Prostate Cancer, and Androgen Receptor Pathway Independent Prostate Cancer.

**DISCUSSION:**

It is clear that new treatment paradigms need to be developed for this diverse group of diseases. The Prouts Neck 2013 Meeting on Prostate Cancer helped to frame the current state of the field and jumpstart ideas for new avenues of treatment. *Prostate 74:314–320, 2014*. © 2013 Wiley Periodicals, Inc.

## INTRODUCTION

After a barren two decades when the only new drug approved for the treatment of castration resistant prostate cancer was docetaxel, the last 3 years has seen a bounty of new agents to help extend the life of men with this lethal disease (see Table1) 1–10. Prostate cancer cell addiction to the androgen receptor (AR) forms the basis both for initial androgen deprivation therapy as well as the new second-generation androgen ablative agents (abiraterone and enzalutamide). Unfortunately, prostate cancers escape these second generation agents and castration resistant prostate cancer (CRPC) remains an incurable disease. Three phenotypes/genotypes of CRPC after treatment with second-generation agents appear to be increasing in prevalence and remain resistant to treatment: NeuroEndocrine Prostate Cancer (NEPC), Persistent AR—Dependent Prostate Cancer (PADPC), and Androgen Receptor Pathway Independent Prostate Cancer (APIPC) 11–14. It is clear that new treatment paradigms need to be developed for this diverse group of diseases.

**I:** Approved Agents for the Treatment of Castrate Resistant Prostate Cancer

Agent	Target	Year approved	Reference
Estramustine	Estrogen mimetic	1981	1
Mitoxantrone	Type II topoisomerase	1996	2
Zoledronic acid	Osteoclast inhibition (adjunctive)	2002	3
Docetaxel	Microtubules	2004	4
Sipuleucel T	Immunomodulation	2010	5
Cabazitaxel	Microtubules	2010	6
Denosumab	RANK ligand (adjunctive)	2010	7
Abiraterone	Androgen synthesis	2011	8
Enzalutamide	Androgen receptor	2012	9
Radium-223	Calcium mimetic	2013	10

The Prouts Neck Meetings on Prostate Cancer began in 1985 through the efforts of the Organ Systems Branch of the National Cancer Institute to stimulate new research and focused around specific questions in prostate tumorigenesis and therapy 15,16. These meetings were unique on many fronts. First, the meetings were relatively small think tanks, composed of around 75 individuals, and divided equally between young investigators and senior investigators. The meeting was organized with short presentations and lengthy discussion times. Over the years, many new concepts related to prostate cancer have resulted from these meetings and the prostate cancer community has sorely missed them since the last one in 2007. Through the support of the Prostate Cancer Foundation, it was decided to re-initiate the Prouts Neck meetings to drive the prostate cancer field forward. The first meeting focused on defining how the field of treatment for metastatic prostate cancer needs to evolve to impact survival and was entitled: “Beyond AR: New Approaches to Treating Metastatic Prostate Cancer”. The meeting focused on delineating some of the current “Big Questions” that need to be addressed by the prostate cancer community as submitted prior to the meeting by the participants.

### What Ultimately Kills People With Metastatic CRPC?

Despite multiple autopsy studies as well as observations by many experienced clinicians the cause of death from metastatic castrate resistant prostate cancer remains poorly defined 17. Although reasons for death such as cancer cachexia and thrombotic events have been documented to account for many deaths, the underlying mediators of these syndromes has not been defined. It is believed that these and other cancer related syndromes are the result of cytokine over production 17–20. Cancer, as it evolves in the host patient over time, acts as multiple endocrine organs, producing local and systemic effects (See Fig. 1) 21–23. A key question is whether identifying and targeting this slurry of soluble factors could decrease the morbidity of cancer.

**Fig I:**
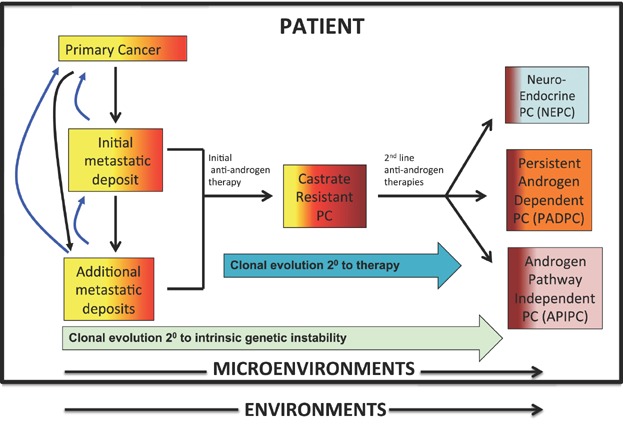
The systems pathobiology of prostate cancer. Prostate cancer develops and evolves as a complex adaptive system in a dynamic manner over time. Different cancer clones (tumor cell heterogeneity) evolve through inherent genomic and epigenomic instability as well as in response to therapeutic pressure. It appears that multiple host cells, including hematopoietic stem cells, mesenchymal stem cells, endothelial progenitors, cancer-associated fibroblasts, and inflammatory mononuclear cells (T-, B-, and monocytes) not only contribute to the pathogenesis of CRPC within the primary and metastatic microenvironments, but also traffic freely between tumor sites 16,17. Three phenotypes/genotypes of CRPC after treatment with second-generation agents appear to be increasing in prevalence and remain resistant to treatment: NeuroEndocrine Prostate Cancer (NEPC), Persistent AR—Dependent Prostate Cancer (PADPC), and Androgen Receptor Pathway Independent Prostate Cancer (APIPC) (9,9a). It is clear that new treatment paradigms, taking into account cancer cell genetic and epigenetic pathways, contributing factors within the microenvironment, and the macroenvironment of the host/patient need to be developed for this diverse group of diseases.

### How Do We Apply Principles of Precision Oncology to the Treatment of CRPC?

Prostate cancer, within a patient, evolves over time as a result of intrinsic genetic instability and as a result of therapeutic pressure (Fig. 1) 16,24–28. While NEPC can arise as an early phenotype independent of castration therapy, all three prostate cancer phenotypes, NEPC, PADPC, and APIPC appear to be increasing in prevalence after treatment with first and second line anti-androgens 16. Identifying these pathways, as well as others, will be dependent on “personalized medicine” or “precision oncology” approaches. Tumor sequencing and other characterization assays will be required to identify the essential combinations of pathways that drive castration resistant prostate cancer. Identifying driver versus passenger mutations will be critical. Serial biopsies of patients (through sequential tissue biopsies or “liquid” circulating tumor cell characterization) will be required to identify the best sequence and/or combined treatment approaches for individual patients.

It is clear, however, that personalized oncology approaches for patients with CRPC are hampered by intra- and inter-tumoral heterogeneity 28–31. Clinical experience shows that within individual patients, some lesions can respond to systemic therapies while others stabilize or progress. Clinically validated biomarkers need to be developed that evaluate this heterogeneity to help prioritize actionable targets for individual patients. Similarly, Can heterogeneity be monitored in real time to more quickly identify emerging cancer clones responsible for therapeutic resistance? It remains unclear if this is possible with currently available technology.

### What Is the Relevance of AR Splice Variants?

Androgen Receptor (AR) splice variants have now been identified and are present in many patients, increasing in frequency after second line anti-androgen therapy 32–36. The importance of these variants, which exhibit loss of the ligand-binding domain, in disease progression, remains undefined. It is likely that these variants contribute to the pathogenesis of PADPC. New agents that target the N-terminal domain of the AR are being developed but their clinical efficacy remains unknown.

### What Is the Role of the Prostate Cancer Stem/Progenitor Cell in Tumor Progression and Resistance?

Identifying a prostate cancer stem cell in mice and men remains elusive 37–39. Several studies have pointed to a basal cell origin for mouse and human prostate cancer but others, at least in the mouse, have demonstrated that both the basal and luminal layers can initiate prostate cancer 38. To date, the most common markers in human to delineate progenitor cells have been a phenotype that includes CD44+/CD133+/ABCG2+/CD24−. Cancer stem cells appear to be rare in primary cancers, in xenografts, and in tissue culture but their number and their role in disease progression remain a mystery 37–40. The continued survival and self-renewal of stem cells/progenitors remains a potential explanation for disease dissemination as well as therapeutic resistance to castration and chemotherapies. The field continues to isolate, characterize, and study how these cells may be generated intrinsically with the cancer cell population as well as how the changing microenvironments of primary and metastatic cancer may influence their behavior.

### How Relevant Is The Tumor Microenvironment in Modulating Cancer Growth and Resistance?

That the tumor microenvironment contributes to tumorigenesis and therapeutic resistance is now widely accepted 41–44. Reactive stroma, including fibroblasts, endothelial cells, osteoblasts, and osteoclasts (in bone), and mesenchymal stem cells all contribute to prostate cancer development and tumorigenesis 22,41–45. Several studies have demonstrated that the stroma can protect cancer cells from radiation and chemotherapy. Since host cells do not exhibit high levels of genetic instability, targeting these facilitative cells present an attractive option for adjunctive therapy 44. Targeting osteoclast function with bisphosphonates is a prototypical example of this in prostate cancer metastasis to bone 42–44. Development of further therapies that target other tumor—host interactions are underway and need to be clinically tested 22,41–44.

### What Is the Evolving Role of Immunotherapy in CRPC?

While in general, the enthusiasm for immunotherapy for prostate cancer utilizing Sipuleucel-T is mixed at best, the recent case report of a patient with metastatic CRPC who achieved a complete and durable biochemical response after treatment with sipuleucel-T while on enzalutamide has been met with much interest 46. In addition, documented responses utilizing the CTLA-4 blocking antibody ipilimumab in prostate cancer as well as melanoma has caught the imagination of the field and suggest that immunotherapy for CRPC is coming of age. Trials need to be performed in combination with antigen presentation strategies such as radiotherapy and cryotherapy 47–49. Moving these therapies into combination studies with androgen ablation or chemotherapy are being tested and to explore the immunologic basis for such a response 47–50. Multiple newer agents, such as those targeting PD-1hold even more promise, however, how these agents provide clinical benefit are not completely understood and biomarkers to predict and monitor response are desperately needed.

### What Is The Evolving Role Of Molecular Imaging in CRPC?

There is wide consensus that the field requires better imaging modalities for metastatic disease, especially in the areas of detecting minimal disease and quantitative responses to therapy 51–53. Bone scans remain qualitative tools and many patients have disease beyond the limits of detection of CT and MRI 54. Several new agents are being developed but the path to approval is difficult and costly. The application of functional imaging to risk-adapted therapy, selection of optimal combination therapies, and prognosis in metastatic prostate cancer does not appear to be likely in the near future. In addition, linking functional imaging as a surrogate biomarker for specific genetic or signaling pathway aberrations is under development but is far from being available for wide use in animal prostate cancer models and in humans. The rational incorporation of novel PET radiotracer(s) and other functional imaging modalities to accelerate and improve therapeutic development is desperately needed.

### Should We Classify Prostate Cancer Progression Based on Molecular Underpinnings of the Disease?

As our understanding of prostate cancer evolution during progression grows, the challenge is to effectively sequence and combine our growing armamentarium of therapeutic agents for maximal patient benefit- the right drugs, in the right combinations, given at the right time; especially by anticipating the need for therapy *before* it is clinically apparent; that is, to move beyond anatomically-based clinical decisions and prognostication to biologically (marker)-driven therapy prediction. Logothetis et al. suggest a molecular classification into four distinct phases of evolution of the disease, based on the underlying molecular mechanisms 55. Stage I, dihydrotestosterone (DHT)-dependent disease that responds to treatment with inhibitors such as dutasteride and finasteride. A subset of these cancers progress to the endocrine-driven stage, or stage II where the tumors are driven by androgens derived from the testes and the gonads, and respond to androgen ablation. Upon androgen deprivation therapy, cancers develop treatment resistance and progress to Stage III, the paracrine, microenvironment-dependent stage of the disease, where factors from the tumor microenvironment in addition to cellular changes from within the tumors drive disease progression. Tumors largely remain androgen signaling driven and respond to next-generation anti-androgens (e.g., abiraterone acetate) and AR inhibitors (e.g., enzalutamide). Cancers that stop responding to these therapies have usually exited the microenvironment-dependent phase and progressed to Stage IV, the Tumor Cell Autonomous phase, the precise mechanisms of development of which aren't clearly understood, however, are likely represented by NEPC, PADPC, and APIPC (Fig. 1).

### What Are the Next Targets for CRPC?

As our knowledge of how CRPC evolves over time continues to grow, new therapeutic targets are being discovered and their value defined (Fig. 1) 56–68. Agents that target DNA repair, altered kinase pathways, and epigenetic pathways are all in development. Agents that target supporting / facilitating host cells of the tumor microenvironment are moving forward. A holistic and integrated approach of altered critical biologic pathways is serving as an impetus for developing new therapeutics and repurposing agents from other diseases 60. Three challenges that continue to stymie progress are the lack of good animal models to test new agents, good adaptive strategies that utilize the current agents, and the lack of our ability to rapidly test combinations of agents in preclinical models, and more importantly in patients 60–63 As our understanding of prostate cancer evolution during progression grows, applying the armamentarium of therapeutic agents in the right sequences in the right combinations at the right time is a major goal in prostate cancer treatment 55.

### What Can We Crowdsource?

The presentations and questions catalyzed the desire for collaborative, transdisciplinary research efforts that will accelerate the search for a cure. One idea that was presented was to gather the unpublished cell line and cell xenograft models that are being utilized within individual labs that could be utilized by others if they simply knew they existed. As a result of the meeting, these resources are being collected and will be publicized through a manuscript as well as the web.

## CONCLUSIONS

The overall consensus was that the meeting, bringing young investigators as well investigators from disparate areas together, resulted in a successful and stimulating exchange of ideas and information. Future investigations on mechanisms of treatment resistance; the role of field cancerization and tumor microenvironment; and determining heterogeneity and its impact on precision medicine are important and warranted. The androgen receptor signaling axis remains a crucial driver of prostate cancer progression and treatment resistance, and newer ways of targeting this axis, as well as PADPC and APIPC, are needed. The vigorous open discussion of unclear and controversial topics helped give everyone a better sense and appreciation of the big unanswered questions and issues in prostate cancer. The 2014 conference will be on the topic: Beyond immune checkpoint blockade: “New Approaches to targeting Host—Tumor Interactions in Prostate Cancer.”
